# Future Applications of Human Milk Reference Values for Nutrients: A Global Resource for Maternal and Child Nutrition Research

**DOI:** 10.1016/j.advnut.2025.100429

**Published:** 2025-08-26

**Authors:** Sophie E Moore, Lindsay H Allen, Gilberto Kac, the MILQ Study Consortium, Lindsay H Allen, Sophie E Moore, Gilberto Kac, Kim F Michaelsen, Christian Mølgaard, M Munirul Islam, Maria Andersson, Setareh Shahab-Ferdows, Sophie H Christensen, Jack I Lewis, Janet M Peerson, Xiuping Tan, Daphna K Dror, Andrew M Doel, Daniela de Barros Mucci, Bruna C Schneider, Farhana Khanam, Adriana Divina de Souza Campos, Gabriela Torres Silva, Fanta Nije, Mehedi Hassan, Amanda C Figueiredo, Daniela Hampel

**Affiliations:** 1 Department of Women & Children’s Health, King’s College London, London, United Kingdom; 2 MRC Unit The Gambia at the London School of Hygiene and Tropical Medicine, Fajara, The Gambia; 3 USDA, ARS Western Human Nutrition Research Center, University of California Davis, Davis, CA, United States; 4 Institute for Global Nutrition, Department of Nutrition, University of California, Davis, CA, United States; 5 Nutritional Epidemiology Observatory, Josué de Castro Nutrition Institute, Federal University of Rio de Janeiro, Rio de Janeiro, Brazil

**Keywords:** breast milk, human milk, lactation, micronutrients, macronutrients, nutrient reference values, recommended nutrient intake, infants, nutrition interventions, supplementation

## Abstract

The World Health Organization recommends exclusive breastfeeding for the first 6 mo of life as human milk is considered to be the optimal form of nutrition to support infant health and development. Human milk provides many nutrient and non-nutrient bioactive compounds to young infants, including micronutrients. In contexts where diets are nutritionally poor, the concentration of micronutrients in human milk is lower, impacting infant's supply. However, understanding when lower values indicate the need for interventions and then evaluating the impact of interventions on maternal nutritional status and milk nutrient concentrations has been challenged by the absence of reliable reference values (RVs) for nutrient concentrations in human milk. The multicenter Mothers, Infants, and Lactation Quality (MILQ) and Early-MILQ studies were developed to establish evidence-based RVs for human milk nutrients. This paper presents and discusses the potential utility of these RVs as an international reference for global maternal and child health research.

**Statement of significance**

Many of the reference values (RVs) for concentrations and intakes of nutrients in human milk developed by the Mothers, Infants and Lactation Quality (MILQ) study differ from those currently used to establish infant requirements and may now be used to update recommended intake estimates. A further application of the MILQ RVs will be to assess population status relative to the percentile distribution and to assess how milk concentrations respond to nutrition intervention programs.

## Introduction

Exclusive breastfeeding (EBF) is recommended for the first 6 mo of life, with continued breastfeeding until the infant is aged ≥2 y. This recommendation from the WHO is built on extensive evidence that supports the importance of EBF for optimal infant health and development [1]. However, the benefits of breastfeeding are known to be wide-reaching, and human milk is an important source of a host of bioactive molecules, including nutrients [2]. The concentration of these bioactives can be impacted by the mother’s diet, health, and the environment in which she lives. It is now established that the concentration of many nutrients in human milk is much lower in contexts where diets are nutritionally poor and, for some nutrients, human milk concentrations are responsive to maternal dietary supplementation [3]. However, until now, there has been no attempt to develop reference values (RVs) for nutrient concentrations in human milk. Therefore, the utility of measuring milk concentrations is restricted, and our understanding of how milk nutrient concentrations, and most notably milk micronutrient concentrations, relate to maternal and infant health outcomes is limited. To address these gaps, the Mothers, Infants, and Lactation Quality (MILQ) and Early-MILQ (E-MILQ) studies were established. Conducted in 4 distinct country settings (Bangladesh, Brazil, Denmark, and The Gambia) and recruiting healthy, well-nourished, unsupplemented mother–infant dyads using matched data and sample collection protocols across settings, the MILQ studies have developed RVs for human milk nutrients [4]. In this paper, we consider how the RVs from MILQ and E-MILQ can be used within the fields of human lactation and nutrition and health research. We focus our discussion here mostly on micronutrients as existing data on the macronutrient content of human milk and its relationships with maternal nutritional status and dietary intake are more extensive.

## Re-evaluation of Nutrient Recommendations for Lactating Women, Infants, and Young Children

Current estimates of most micronutrient requirements for lactating women, infants, and young children are based on the mean concentrations in a small number of milk samples from studies with very limited information available on study design, maternal diet and nutritional status, or milk sampling protocols [5]. Typically, laboratory methods used for measuring concentrations were not validated for the analysis of milk, and fewer micronutrients could be measured before the availability of mass spectrometry [6]. Furthermore, estimates were derived from samples collected throughout lactation, so it was not possible to adjust for the changes in concentration that occur at different stages. These mean values, all multiplied by the same estimate of daily milk volume (0.78 L), constitute the current adequate intakes (AIs) for infants and, for most nutrients, the additional maternal nutrient requirements for lactation [7]. The estimate of daily milk volume was generated as the mean volume of milk intake reported from data available at the time, assessed by test weighing [7]. Although many of the original studies were conducted over 40 y ago, and restricted to test weighing, this estimate is remarkably consistent with the mean value derived from MILQ across the period of lactation investigated.

The RVs and milk volumes generated from the MILQ study can now be used to construct improved intake recommendations for infants, such as estimated average requirements with which it is possible to determine the percent of infants in a population group with inadequate intakes. One of the critical and novel findings of the MILQ study is that milk volume is relatively weakly correlated with the micronutrient concentrations measured in milk samples collected during the window of milk volume assessment, meaning that both milk nutrient concentration and the volume of milk an infant consumes impact total micronutrient intake, but these are not often measured together. This represents a challenge for the human milk field as, in addition to the laboratory resources required to measure nutrient concentrations, the assessment of human milk intake is resource heavy, requiring repeat measures over a 24-h period (test weighing) or isotope administration and sample collection over a 14-d period, followed by sample analysis (isotope dilution) [8,9]. It is therefore not practical to recommend the assessment of milk intake in every study where milk nutrient concentration is measured. For EBF infants, key determinants of milk intake are infant age and body weight [8,10]. Future considerations of nutrient intakes should therefore take into account these parameters and the importance of having recommendations that reflect infant dynamics.

Current AI estimates are also often extrapolated to derive recommended nutrient intakes for young children, which are the basis for estimating nutrient gaps and requirements for complementary feeding. The median concentration of some micronutrients measured in the MILQ study (in infants aged 1–6 mo) is lower than the value used currently by the National Academy of Medicine (NAM, formerly Institute of Medicine, IOM) to set recommendations (set to reflect infants aged 0–6 mo but, for some nutrients, values from early in lactation were intentionally chosen when concentrations are higher) [7]. If used to estimate nutrient intakes, these lower values may lead not only to changes in the recommended levels of nutrients in human milk substitutes but also to changes in the formulation of complementary foods [11].

## Evaluation of the Impact of Nutrition-Specific and Nutrition-Sensitive Interventions on Milk Composition and Nutrient Status

The United Nations Sustainable Development Goal number 2 is to end hunger and achieve food security by 2030. Despite some gains, the latest Global Nutrition Report highlights widespread food insecurity and resulting hunger [12]. Food and nutrition insecurity directly impacts diet quality and is strongly linked to micronutrient deficiencies. Global, national, and regional efforts focused on interventions to improve access to food are considerable, and program evaluation is essential to ensure these are effective.

Strategies to improve maternal and child nutrition can be broadly split into interventions that are nutrition specific (e.g. interventions that directly address the immediate causes of undernutrition) and those that are nutrition-sensitive (e.g. interventions that address the underlying causes of undernutrition) [13]. Examples of nutrition-specific interventions include multiple micronutrient supplementation during the antenatal and postnatal period and national food fortification programs, e.g. industrial fortification of wheat flour with micronutrients. Examples of nutrition-sensitive interventions include Water, Hygiene, and Sanitation activities [14] and agricultural programs, such as livestock breeding [15]. Many of these programs have been tested in trials on maternal and child health, with an increasing number of them including the collection and analysis of human milk samples as part of their evaluation. In previous studies of population groups consuming diets with inadequate intake of many micronutrients, we have reported low milk concentrations of those micronutrients and the positive impact of maternal supplementation [16,17] and flour fortification [18]. However, until now, the ability to infer impact is limited to group comparisons (e.g. judging if the intervention has outperformed the control) or comparisons against existing AIs, which, for the reasons outlined previously, may not provide a reliable estimate of true impact as it relates to maternal or infant status.

The RVs generated by MILQ provide an updated, internationally relevant resource against which to evaluate the true impact of nutrition interventions. The MILQ RVs can contribute to the evaluation of both efficacy and effectiveness across national and global programs, with the aim of understanding which programs are likely to yield the greatest impact on maternal, infant and child health outcomes. A specific example is the evaluation of the multiple micronutrient formulation (the United Nations International Multiple Micronutrient Preparation, “UNIMMAP”) currently recommended by WHO for use in the context of rigorous research [19]. UNIMMAP is both efficacious and effective in pregnancy trials, positively impacting birth outcomes [20]. However, limited research has effectively evaluated the impact of the UNIMMAP formulation on infant outcomes, including infant nutritional status. Several trials (completed and ongoing) have collected human milk samples following supplementation in pregnancy (and in a few cases, continuing into lactation). The impact of intervention on micronutrient concentrations in human milk has been evaluated, with inferences drawn over the impact. A systematic review of the impact of all trials on micronutrient status is currently ongoing (https://www.crd.york.ac.uk/prospero/display_record.php?ID=CRD42023448676). Comparing the results obtained against MILQ RVs will enable an evaluation of the interventions with respect to nutrient adequacy; all previous analyses have been limited to looking at the effect size of the response.

## Biomarkers for Maternal and Infant Nutritional Status

Micronutrient deficiencies exacerbate a broad array of adverse health and functional outcomes, with women of reproductive age and infants and young children, especially in low-resource settings, being among the most at-risk groups. However, the true extent of the problem is unknown mainly because of the lack of reliable population-representative biomarker data on micronutrient status [21]. This has widespread consequences, including the common failure to consider micronutrient deficiencies, often referred to as “hidden hunger” when considering the global burden of malnutrition [22].

There are several reasons for this lack of information, which Brown et al. [21] have discussed at length. Key limitations are collecting, processing, and storing blood samples for biomarker assessment. The inclusion of lactating women in national surveys with the collection of human milk in at least a subsample represents an opportunity to contribute to a more complete understanding of population-level deficiencies. Hand expression of a small volume of milk into storage tubes avoids the complexities of venous blood sampling and the need to process samples on site (e.g. separation of whole blood). Sample storage and analytical facilities are still required, but with developments in the ability to measure multiple micronutrients in a single sample [23] and innovations around the use of dried milk spots [24], the use of human milk for the assessment of micronutrient status should be considered more widely. Although the deliberate selection of well-nourished women in the MILQ study reduced our ability to demonstrate intersite differences in composition caused by maternal deficiencies, lower vitamin D in milk from urban Bangladesh suggests that mothers were lacking in this nutrient. This will be confirmed in pending analyses of vitamin D status biomarkers in blood from both the mothers and the infants, but highlights the potential utility of human milk RVs in population surveillance.

The MILQ study will also yield nutritional status biomarker RVs for postpartum lactating women and their infants, through the analysis of biobanked blood and urine samples. Although human milk may be used to assess maternal micronutrient status and infer infant intake for some nutrients, it may not be suitable for all. Vitamins (except folate) in milk are more affected by maternal intake than minerals (except iodine and selenium), and milk levels of vitamins are more affected by maternal supplementation and fortification than by usual dietary intake [3].

## Strengths and Limitations

The key strengths of the MILQ study, as outlined in the introductory paper to this series [25], relate to the matched protocols employed across the four countries to recruit healthy, well-nourished women, continuous data across the first 8.5 months of lactation, and dedicated protocols for sample collection and processing, and the use of analytical methods developed to assess nutrient concentrations in the matrix of human milk. This latter strength may also present a challenge for the nutrition and public health community as access to the required laboratory platforms, such as mass spectrometers, may be limited, a challenge also discussed in the previously highlighted paper from Brown et al. [21].

In addition to these strengths, the data obtained reveal several limitations that must be considered when using the RVs. First, for some nutrients, apparent differences were found in the nutrient concentrations in human milk between sites despite the purposive inclusion of well-nourished women, most notably for vitamin D in the samples collected from Bangladesh, and iodine across sites. Furthermore, for some nutrients (e.g. vitamin B_12_), a wider-than-expected variation was seen in milk concentrations among participating mothers. Rather than restrict the dataset to a more limited set of values, we have included all the data obtained except for vitamin D in Bangladesh. Eligibility criteria and measurement of biomarkers in the MILQ study suggest that these represent acceptable variations in a healthy and diverse population of lactating women. Finally, the MILQ RVs reflect milk nutrients from healthy, term infants. Although they can be used to make comparisons with specific clinical groups (e.g. preterm infants), it will not be possible to use them to determine the nutrient needs of such groups.

## Conclusions

The newly created MILQ RVs for human milk provide novel and comprehensive data on milk nutrient concentrations and will serve as an international reference against which to compare milk macro- and micronutrient concentrations and estimated infant intakes. This will help to identify groups or populations of infants (and mothers) at risk of deficiency, supporting the design and implementation of targeted interventions. Furthermore, where human milk sampling and measuring are integrated into studies and surveys, the response to programs, such as national fortification programs, can be monitored and evaluated. Finally, by eventually integrating the comprehensive data available on maternal and infant status against human milk concentrations, the MILQ RVs will help determine what level of adequacy of maternal status corresponds to milk adequacy and vice versa; this latter analysis will enable evaluation of the utility of biomarkers measured in human milk samples for use in population surveys. Together, this will support intervention, program, and policy planning focused on maternal and infant nutrition. Decisions can be made locally about how to use the milk concentration percentiles, for example the acceptability of a population group, or a subsample of a population, being at the 20th percentile of the RV for 1 or more vitamins.

Beyond nutrients, the samples and data collected as part of the MILQ study are being used to evaluate how more novel components of human milk relate to infant growth. These include human milk oligosaccharides, the milk transcriptome, proteome, and metabolome, maternal genetics, and the infant gut microbiome. Employing a multiomic or systems biology approach, this work will help develop our understanding of how these components of human milk work individually or jointly to influence infant growth and associated outcomes. Together with the nutrient RV data already generated, the data and biobank generated through the MILQ collaboration will form an essential resource for supporting global population work on human lactation in the future.

## Author contributions

The authors’ responsibilities were as follows – SEM, LHA, GK: designed the study and wrote the paper; SEM: had primary responsibility for the final content; and all authors: read and approved the final manuscript.

## Data availability

Data described in the manuscript, code book, and analytic code will be made available upon request pending application and approval.

## Funding

Supported by the Gates Foundation (OPP1148405/INV-002300, OPP1061055) and USDA intramural funds (2023-51530-025-00D). Sophie E. Moore is supported by a Wellcome Trust Senior Research Fellowship (220225/Z/20/A).

## Conflict of interest

The authors report no conflicts of interest.
